# The Maternal Serological Response to Intrauterine *Ureaplasma* sp. Infection and Prediction of Risk of Pre-Term Birth

**DOI:** 10.3389/fimmu.2014.00624

**Published:** 2014-12-09

**Authors:** Demelza J. Ireland, Jeffrey A. Keelan

**Affiliations:** ^1^School of Women’s and Infants’ Health, The University of Western Australia, Perth, WA, Australia

**Keywords:** antibody, immune response, intrauterine infection, pre-term birth, predictive marker, *Ureaplasma* spp.

## Abstract

Pre-term birth (PTB) associated with intrauterine infection and inflammation (IUI) is the major cause of early PTB less than 32 weeks of gestation. *Ureaplasma* spp. are common commensals of the urogenital tract in pregnancy and are the most commonly identified microorganisms in amniotic fluid of pre-term pregnancies. While we have an understanding of the causal relationship between intra-amniotic infection, inflammation and PTB, we are still unable to explain why vaginal *Ureaplasma* sp. colonization is tolerated in some women but causes PTB in others. It is now known that placental tissues are frequently colonized by bacteria even in apparently healthy pregnancies delivered at term; usually this occurs in the absence of a significant local inflammatory response. It appears, therefore, that the site, nature, and magnitude of the immune response to infiltrating microorganisms are key in determining pregnancy outcome. Some evidence exists that the maternal serological response to *Ureaplasma* sp. colonization may be predictive of adverse pregnancy outcome, although issues such as the importance of virulence factors (serovars) and the timing, magnitude, and functional consequences of the immune response await clarification. This mini-review discusses the evidence linking the maternal immune response to risk of PTB and the potential applications of maternal serological analysis for predicting obstetric outcome.

## Introduction

It is estimated globally that approximately 12 million babies are born pre-term each year, making the prevention of pre-term birth (PTB) one of the highest priorities for international obstetric research (1). Intrauterine infection (IUI) and subsequent inflammation of the extra-placental membranes (chorioamnionitis) accounts for approximately 40% of all spontaneous PTBs and is the major cause of early PTB (<32 weeks of gestation). IUI is typically “silent” (undiagnosed) until the onset of pre-term labor at which point it is often too late for treatment as chorioamnionitis is well established, the risk of fetal inflammatory response syndrome (FIRS) is high (2), and tocolysis is ineffective. Identifying women at risk of infection-associated PTB sufficiently early in pregnancy to allow therapeutic intervention would be a significant advance in the prevention of PTB.

Traditional thinking associates IUI with the ascension of bacteria from cervicovaginal fluid, resulting in intra-amniotic infection and immune stimulation within the otherwise sterile intrauterine environment (3). It is now clear, however, that the placenta and extra-placental membranes can no longer be considered strictly sterile (4, 5). Instead, they are home to a unique microbiome of non-pathogenic commensals; the presence of which is normal and not associated with early delivery or adverse pregnancy outcomes (4, 6). Histological and immunological analysis of intrauterine tissues and fluids suggests that the nature and magnitude of inflammatory response associated with bacterial colonization may be key in determining obstetric outcome (6–8).

Recent placental microbiological studies have reignited debate regarding the role of differential virulence (9), poly-microbial interactions (10), host genetics (11), and immune factors (12) in determining obstetric outcome. While attention has primarily been placed on defining the local immune response to bacteria within placental tissues, the role and significance of the maternal systemic immune response to the infection has been largely neglected. Yet, studies conducted at the end of the last century strongly suggested that the maternal immune response to commensal microorganisms found in the urogenital tract in pregnancy – in particular the *Ureaplasma* species – may provide us with important clues as to why some women are at risk of adverse pregnancy outcomes while the majority are not.

In this mini-review, we discuss in detail the somewhat contradictory evidence relating to the presence and nature of maternal antibodies to *Ureaplasma* sp. and their significance in determining and predicting obstetric outcome. A specific focus is placed on the potential clinical utility of serological analysis in the identification of women at elevated risk of PTB.

## *Ureaplasma* and PTB

*Ureaplasma* spp. are generally considered commensal microorganisms (13–16) and are classified into two species and 14 distinct serovars (SV). SV1, SV3, SV6, and SV14 belong to *U. parvum* species and the remaining ten SV to *U. urealyticum* (17). *Ureaplasma* sp. commonly colonize the urogenital tract of both males and females (18, 19). Vaginal colonization rates can vary greatly in non-pregnant women (up to 70%) (20, 21) and women with uncomplicated pregnancies [2.7–70%; reviewed in Ref. (22)]. *Ureaplasma* spp. are some of the most frequently identified microorganisms in placental tissues and amniotic fluids (AF) from pre-term deliveries (23–25). Colonization of the placenta with *Ureaplasma* sp. has been demonstrated to be an independent risk factor for chorioamnionitis [odds ratio (OR), 11.27; 95% CI, 5.09–24.98] (7). Detection rates in AF vary from 0% to 19% in early mid-trimester (26–28), to 2–80% at pre-term labor (26, 29) and 18–100% with pre-labor premature rupture of membranes (PPROM) (26, 30). A meta-analysis of 22 studies found a significant association between the presence of *Ureaplasma* sp. in the vagina and AF with PTB (22). However, *Ureaplasma* sp. colonization in the urogenital tract and AF is a relatively common finding in pregnant women and alone it is not sufficiently predictive of PTB to be clinically useful (8).

**Figure 1:**
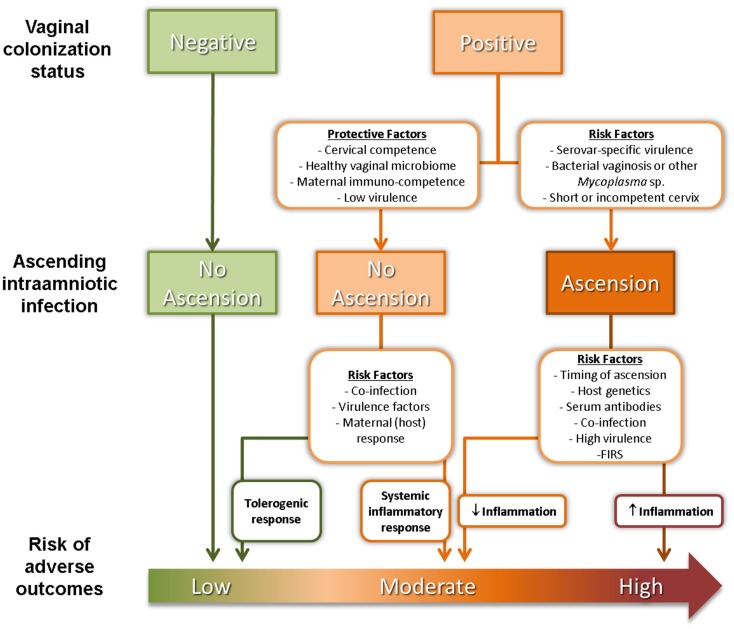
**Factors affecting ascension of vaginal *Ureaplasma* sp. and risk of adverse pregnancy outcomes**.

The reasons why commensal *Ureaplasma* sp. cause ascending IUI leading to PTB in only a subset of women are still unknown but are likely to be complex and multifactorial (Figure 1). SV-specific virulence has been proposed as an important determinant of risk of adverse outcome. Of the two *Ureaplasma* species, *U. parvum* is the most commonly isolated species from clinical samples (31), with two of its SV – SV3 and SV6 – associated with worse pregnancy outcomes (32–34). Unfortunately, most studies of IUI do not differentiate between *Ureaplasma* SV so data on the relationship between SV prevalence and risk are lacking. Poly-microbial interactions may also be significant as diagnosis of abnormal bacterial flora or bacterial vaginosis (BV) (i.e., a high Nugent score) is also a risk factor for PTB (35), independent of *Ureaplasma* sp. colonization status. It is worth noting that poly-microbial colonization of the amniotic cavity is common in pre-term deliveries, with around half of all infected AF containing two or more microorganisms (23). Co-colonization of the vagina with a *Ureaplasma* sp. and another genital *Mycoplasma* sp. has been reported to be associated with more severe adverse pregnancy outcomes compared to colonization with a single organism (36). Competence of the cervical barrier to microbial ascension is also likely to play a role in determining risk of PTB. A short cervix in pregnancy has been associated with increased risks of microbial colonization (37) and spontaneous PTB (38, 39).

Finally, maternal/fetal immunological tolerance or competence is a likely determinant of obstetric outcome associated with colonization by *Ureaplasma* sp. and other microorganisms. Surface-exposed lipoproteins of *Ureaplasma* sp. activate the pro-inflammatory transcription factor NF-κB through TLR ligation (40, 41), although exposure of intrauterine tissues to *Ureaplasma* sp. does not generally trigger a robust inflammatory response. Nevertheless, intra-amniotic infection with *U. parvum* has been causally linked to chorioamnionitis, FIRS and PTB in animal models (42–44). This is consistent with observations from clinical studies showing that chorioamnionitis/funisitis is more likely in pregnancies infected with *Ureaplasma* sp. as opposed to other bacteria (7). On the other hand, most women with vaginal colonization and local inflammatory response will not deliver preterm (24, 45). Combs, Gravett (8), proposed a model of IUI comprising of five subgroups: (1) microbial colonization plus inflammation (AF IL-6 >11.3 ng/ml); (2) severe inflammation with no detectable microorganisms (AF IL-6 >11.3 ng/ml); (3) mild inflammation with no detectable microorganisms (AF IL-6 of 2.6–11.2 ng/ml); (4) microbial colonization with no inflammation; and (5) absence of infection or inflammation. This more complicated scenario highlights the need to accurately stratify women at risk of PTB associated with IUI.

## Maternal Antibodies Against *Ureaplasma* for the Prediction of PTB

### Anti-*Ureaplasma* serum antibodies in pathogenesis or protection from disease

Specific antibodies may be produced in response to a bacterial infection and detected in the serum (seropositivity). Antibodies act to neutralize bacterial toxins, facilitate opsonization, and together with the complement system work toward clearance of the infection. Antibodies generated against surface-exposed *Ureaplasma* sp. epitopes have been identified and shown to inhibit *Ureaplasma* sp. metabolism (46) and support complement mediated *Ureaplasma* sp. clearance (47) suggestive of a protective effect. Interestingly, individuals with the greatest number/intensity of anti-*Ureaplasma* antibody bands by immunoblot appeared to have higher *Ureaplasma* sp. killing ability (47). These data are in contrast with other studies which report that the presence of anti-*Ureaplasma* antibodies in colonized pregnant women are associated with worse pregnancy outcome [reviewed in Ref. (26); see Anti-*Ureaplasma* Antibodies as Biomarkers for IUI and PTB below]. In these circumstances, it is likely that seropositivity is pathologic rather than protective, possibly acting via an exacerbation of inflammatory processes triggering PTB (48). This concept is supported by data from a pregnant sheep model of intra-amniotic *Ureaplasma* sp. infection, in which increased intrauterine inflammation was detected in sheep in which maternal anti-*Ureaplasma* IgG was also detected (9). Alternatively, it may be the absence of a specific antibody that confers protection from PTB. The key to unraveling this confusion is the identification of antigen–antibody characteristics associated with a given pregnancy outcome to determine whether it is antibody abundance (i.e., titer), nature of the target (i.e., virulence factor), onset of response (time of exposure), or capacity to induce microbial lysis and destruction that is associated with risk of PTB.

### Anti-*Ureaplasma* antibodies as biomarkers for IUI and PTB

**Figure 2:**
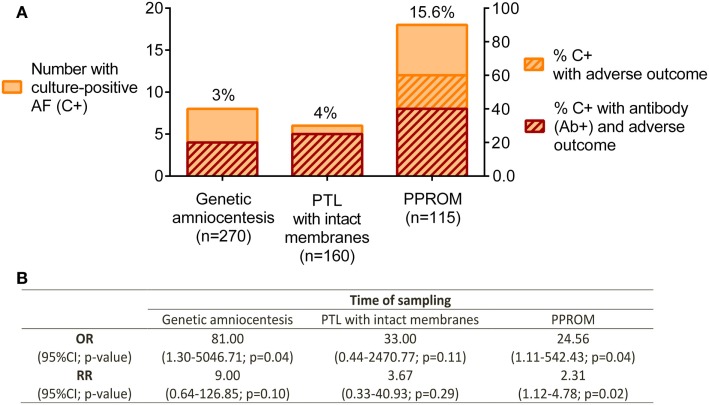
**Pregnancy outcome in relation to culture-positive (C+) AF and anti-*Ureaplasma* antibodies (Ab+) in maternal serum**. **(A)** *Ureaplasma* sp. detected by culture technique. Antibodies determined by ELISA and semi-quantitation of antibody titer; adverse pregnancy outcome defined as fetal loss, stillbirth, pre-term delivery, and low birth weight. Genetic amniocentesis was performed at 16–20 weeks of gestation; no gestational age provided for the PTL and PPROM groups. **(B)** Odds ratios (OR) and relative risks (RR) for adverse pregnancy outcome in culture-positive/antibody-positive (C^+^Ab^+^) vs. C^+^Ab^−^ were calculated for each group. Data extracted and adapted from Horowitz et al. (26).

In the 1980–1990s, several research groups investigated the antibody response to *Ureaplasma* sp. as a biomarker for intra-amniotic or urogenital infections (49–51). A review on the topic of assessment of antibodies for identification of intra-amniotic infection with *Ureaplasma* sp. during pregnancy was published in 1994 by Shulamith Horowitz (26). He reviewed the literature and correlated cervical and intra-amniotic *Ureaplasma* sp. colonization rates with the presence of antibody and pregnancy outcome. Specifically, he found that a greater percentage of women had adverse pregnancy outcomes if they had positive *Ureaplasma* sp. AF cultures and elevated serum anti-*Ureaplasma* antibody titers at either genetic amniocentesis, or pre-term labor with/without PPROM (defined as fetal loss, stillbirth, pre-term delivery, or low birth weight) (Figure 2A) (26). Odds ratios and relative risks for adverse pregnancy outcome in culture-positive (C+) /antibody-positive (Ab+) women vs. C+/antibody-negative (Ab−) women were calculated based on these data and are presented in Figure 2B. Despite some discrepancy between studies, the detection of anti-*Ureaplasma* IgG in maternal sera together with AF colonization was predictive of an increased risk of developing pregnancy complications [OR: 81.00 at genetic amniocentesis (*p* = 0.04); OR: 24.56 (*p* = 0.04); and RR: 2.31 (*p* = 0.02) at PPROM]. Despite these encouraging findings, this line of research has not matured over the proceeding decades for the reasons outline below.

### Factors that have limited the clinical translation of maternal antibody detection for prediction of PTB

Unfortunately, routine amniocentesis for the purpose of diagnosing intra-amniotic colonization with *Ureaplasma* sp. is not clinically feasible due to the procedure-associated risk of spontaneous miscarriage (RR 1.60; 95% CI 1.02–2.52) (52). Furthermore, the rates of *Ureaplasma* sp. colonization of AF in mid pregnancy are actually very low (22, 26, 27). As such, consideration has been given to the ability of maternal antibodies alone to stratify pregnant women for risk of PTB.

### Seroconversion in response to *Ureaplasma* is common

Early studies varied in their reported rates of seroconversion. ELISA based studies reported anti-*Ureaplasma* IgG seropositivity rates of 50–85% in culture-positive individuals and 6–15% in culture-negative individuals (46, 53, 54). However, these studies reported positive cutoffs based on maximum detection in culture-negative cohorts, assuming culture-negative individuals to be seronegative (53, 54). Others have since shown by immunoblot that seroconversion is more common, with greater than 80% of sera from healthy non-pregnant individuals (47) recognizing at least one *Ureaplasma* sp. antigen (IgG response). As the seroconversion rate is high in both colonized and non-colonized individuals, several studies used a method of serum dilution for assigning positivity to sera with elevated titers (46, 55). It is still unclear whether differences between patients with slightly elevated vs. very high levels of antibodies are clinically significant. As colonization is so common, it is most likely that seropositivity in non-colonized individuals represents a persistent antibody to a cleared infection. Nevertheless, high seroconversion rates mean that detection of IgG antibody responses to whole cell lysates or isolated membrane antigens is unlikely to be clinically useful.

### Serovar-specific *Ureaplasma* antigens and cross-reactivity

*U. parvum* SV3 and SV6 are most commonly associated with worse pregnancy outcome (32–34). The possibility of a SV-specific antibody response to *Ureaplasma* sp. was proposed following the identification of apparent SV-specific antigens. For example, an 85 kDa protein in SV1, a 71 kDa protein from SV3, 61 kDa and 55 kDa proteins from SV6, and an 88 kDa protein from SV14 have all been identified (56). However, there is extensive antigen cross-reactivity among all SV with the evidence for SV specificity strongest in individual cases. It is likely that the multiple-banded antigen (MBA) is a key virulence factor for *Ureaplasma* sp. (57), containing both SV-specific and conserved/cross-reactive epitopes (58). The MBA, a surface-expressed diacylated lipoprotein, is recognized by TLRs 1, 2, 6 (40), and 9 (41) and has a molecular weight of approximately 70–90 kDa (59), but migrates as a symmetrical ladder pattern between 55 and 75 kDa on immunoblot due to conformational folding (58). The *mba* gene exists as a single copy within the *Ureaplasma* sp. genome (60). The precise immunogenic MBA epitopes are still unknown but are considered to be within the size-variable and surface-exposed C-terminal region (58). Mutations in the *mba* gene have been proposed to play an important role in helping the organism evade the host immune system (60) with multiple mechanisms proposed (58–61).

Serum from *Ureaplasma* sp. colonized individuals detect multiple and likely closely related antigens forming the ladder seen on immunoblots. The identity of specific immunodominant epitopes and their role in differential pathogenesis in pregnancy remains unclear and is further complicated by the variability of the MBA. A number of cases where mother-fetus dyads appeared to be colonized with different SV and generated different antibody responses (as determined by ELISA) have been observed (62). It needs to be remembered that *Ureaplasma* spp. are fastidious organisms, and without careful collection and appropriate culture protocols their presence may have been missed (63). Molecular methods, in combination with routine culture, are now preferred for the detection and speciation of *Ureaplasma* sp. in clinical samples (64).

The lack of robust and reliable techniques to differentiate between SV, the diversity of responses among individuals and the cross-reactivity between SV have significantly compromised the ability to use antibody responses beyond the simple determination of seroconversion. Clarification of the relationship between SV-related antigens, microbial virulence, and differential systemic immune responses will be required to resolve the present impasse.

### Fetal antibody responses to *Ureaplasma* and obstetric outcome

While our emphasis has been on the maternal immune response as a determinant of obstetric outcome, fetal systemic responses can undoubtedly contribute to progression to PTB (65). *In utero* exposure to *Ureaplasma* sp. is commonly associated with increased incidence of neonatal complications including bronchopulmonary dysplasia, intraventricular hemorrhage, necrotizing enterocolitis, and pneumonia (66–69). Fetal production of antibody against *Ureaplasma* sp. has been reported (70–72), with antibody detected at birth in neonates as young as 22–27 weeks of gestation. Moreover, antibody titer appears to correlate negatively with neonatal outcome. IgM has been detected in the fetus/neonate consistent with an initial stage of immune recognition (62). In the sheep model, anti-*Ureaplasma* IgG has also been detected in fetal serum, with one case reported where the antibodies in maternal and fetal sera recognized different antigens (9). Furthermore, fetuses which developed systemic *Ureaplasma* sp. infection [culture-positive cerebrospinal fluid (CSF)] were found in ewes with the lowest numbers of MBA variants in AF and the highest monocyte count in the chorioamnion (61). Thus, monitoring maternal responses alone may fail to identify the fetuses at most risk *in utero*. Unfortunately, acquiring blood antenatally for determination of fetal seroconversion is not a practical approach for assessing risk of PTB.

## Cellular Responses to *Ureaplasma*

*Ureaplasma* sp. have been shown to stimulate pro-inflammatory responses in fetal membranes (73), choriodecidual explants (74), and preterm and term cord blood (75). However, studies of white blood cell responses to *Ureaplasma* sp. in pregnancy have not proven to be useful to date, with no differences in responsiveness observed in leukocytes from women at risk of PTB compared to those with normal pregnancies (12). Reyes et al. explained differential pathogenesis of *Ureaplasma* sp. in urinary tract infections (UTIs) by demonstrating the presence of two distinct immune cell profiles (76). Asymptomatic UTIs were characterized by minimal monocytic and lymphocytic infiltration, less tissue damage and increased IFN-γ, while complicated UTIs were associated with greater concentrations of pro-inflammatory cytokines, extensive inflammation and predominantly a neutrophilic response. No comparable data from pregnant women have been published to date. Recently, unconventional lipid antigens such as those present in *Ureaplasma* sp. (9, 57), and microbe-derived vitamin B metabolites (77), have also been shown to stimulate T cell responses vital for microbial clearance (78, 79). However, the role of these unconventional T cells in the response to *Ureaplasma* sp. in the context of pregnancy immunity and PTB risk remains unknown and the role of the systemic immune cell responses to *Ureaplasma* sp. exposure in pregnancy remains relatively unexplored.

## Concluding Remarks

Despite advances in our understanding of the causal relationships between intrauterine infection and PTB, major research questions remain unanswered: is there a subpopulation of patients in which *Ureaplasma* sp. colonization correlates with worse disease outcome? Does the site of colonization determine disease outcome? Are there pathogenic and non-pathogenic SV determining disease outcome? Is there a specific aspect of the maternal immune response to *Ureaplasma* sp. infection that influences risk of PTB?

Research on specific *Ureaplasma* sp. SV and virulence factors have failed to yield conclusive results. Despite the earlier clinical promise of antibody-based predictive approaches (26, 62), the assessment of maternal or fetal seropositivity to identify women for prophylactic treatment has not made it into clinical practice. It is unclear when and where exposure to *Ureaplasma* sp. antigens takes place in pregnancy and when/how commensal colonization becomes an infection. As such, it is difficult to define the role of antibodies in pathogenesis. Antibody cross-reactivity among the *Ureaplasma* SV and lack of stringent proof of epitope specificity have also limited attempts to use an individual’s antibody response to make a SV-specific diagnosis of infection or predict an outcome.

Before an antibody-based immunological test can be considered as part of a routine antenatal screen, future studies must address technical and scientific issues surrounding the detection and antigen characterization of antibodies to *Ureaplasma* sp. (41, 46, 47, 54, 58). Key issues to be addressed include: (i) need for the identification of the colonizing SV; (ii) requirement for the detection of a SV-specific or global antibody response; (iii) characterization of immunodominant epitopes; (iv) defining the difference between commensal colonization and infection; (v) determining when and where *Ureaplasma* sp. are first immunologically detected; and (vi) characterizing the kinetics and magnitude of the antibody response in relation to pregnancy outcome. Based on current knowledge, it seems that the detection and measurement of uncharacterized maternal antibodies against *Ureaplasma* sp. has limited predictive value for identifying women at elevated risk of infection-driven PTB.

## Conflict of Interest Statement

The authors declare that the research was conducted in the absence of any commercial or financial relationships that could be construed as a potential conflict of interest.
